# MRI and pathology comparisons in Rasmussen’s encephalitis: a multi-institutional examination of hemispherotomy outcomes relative to imaging and histological severity

**DOI:** 10.1007/s00381-024-06353-4

**Published:** 2024-03-15

**Authors:** Alexander Doherty, Kathleen Knudson, Christine Fuller, James L. Leach, Anthony C. Wang, Neena Marupudi, Rowland H. Han, Stuart Tomko, Jeff Ojemann, Matthew D. Smyth, Francesco Mangano, Jesse Skoch

**Affiliations:** 1 University of Cincinnati College of Medicine, Cincinnati, OH USA; 2 Division of Pediatric Neurosurgery, Cincinnati Children’s Hospital and Medical Center, Cincinnati, OH USA; 3 Department of Neurosurgery and Spine, ECU Health, Greenville, NC USA; 4 Pathology, SUNY Upstate Medical University, Syracuse, NY USA; 5 Radiology, Cincinnati Childrens Hospital and Medical Center, Cincinnati, OH USA; 6 Department of Neurosurgery, Mattel Children’s Hospital, University of California Los Angeles, Los Angeles, CA USA; 7 Department of Neurosurgery, Michigan Medicine, Ann Arbor, MI USA; 8 Department of Neurological Surgery, Washington University School of Medicine, St. Louis, MO USA; 9 Neurology, Washington University, St. Louis Children’s Hospital, St. Louis, MO USA; 10 Department of Neurosurgery, Seattle Childrens Hospital, Seattle, WA USA; 11 Johns Hopkins All Children’s Hospital, St. Petersburg, FL USA

**Keywords:** Rasmussen’s encephalitis, Hemispherotomy, Seizure freedom, HOPS

## Abstract

**Purpose:**

Rasmussen encephalitis (RE) is a very rare chronic neurological disorder of unilateral inflammation of the cerebral cortex. Hemispherotomy provides the best chance at achieving seizure freedom in RE patients, but with significant risks and variable long-term outcomes. The goal of this study is to utilize our multicenter pediatric cohort to characterize if differences in pathology and/or imaging characterization of RE may provide a window into post-operative seizure outcomes, which in turn could guide decision-making for parents and healthcare providers.

**Methods:**

This multi-institutional retrospective review of medical record, imaging, and pathology samples was approved by each individual institution’s review board. Data was collected from all known pediatric cases of peri-insular functional hemispherotomy from the earliest available electronic medical records. Mean follow-up time was 4.9 years. Clinical outcomes were measured by last follow-up visit using both Engel and ILAE scoring systems. Relationships between categorical and continuous variables were analyzed with Pearson correlation values.

**Results:**

Twenty-seven patients met study criteria. No statistically significant correlations existed between patient imaging and pathology data. Pathology stage, MRI brain imaging stages, and a combined assessment of pathology and imaging stages showed no statistically significant correlation to post-operative seizure freedom rates. Hemispherectomy Outcome Prediction Scale scoring demonstrated seizure freedom in only 71% of patients receiving a score of 1 and 36% of patients receiving a score of 2 which were substantially lower than predicted.

**Conclusions:**

Our analysis did not find evidence for either independent or combined analysis of imaging and pathology staging being predictive for post peri-insular hemispherotomy seizure outcomes, prompting the need for other biomarkers to be explored. Our data stands in contrast to the recently proposed Hemispherectomy Outcome Prediction Scale and does not externally validate this metric for an RE cohort.

## Introduction

Rasmussen’s encephalitis (RE) is a very rare immune-mediated chronic neurological disorder of unilateral inflammation of the cerebral cortex [1], impacting 1.7–2.4 per 10 million children under the age of 18 [2–4]. The exact pathophysiologic mechanism is unknown, but previous research has indicated the disease is likely driven by a T-cell response with a potential contribution from autoantibodies [1, 5]. Typical initial symptomatology for RE includes seizures originating from one cerebral hemisphere, with 40% developing hemiparesis within a year of symptom onset [6]. Epilepsia partialis continua (EPC), prolonged repetitive arrhythmic focal (typically motor) seizures, is also commonly observed (50–81%) [7, 8]. Early MRI features have also been noted, such as focal cortical atrophy, white matter hyperintensity, and caudate head nucleus atrophy. These typically occur within approximately 4 months of seizure onset [9].

Many treatment options have been attempted for those with RE. These include anti-seizure medications, as well as antiviral and immunosuppressive therapies [10–13]. Unfortunately, the epilepsy becomes refractory to medicinal interventions within months of onset [9]. Disconnective surgical hemispherotomy provides the best chance at achieving seizure freedom in RE patients [1, 14], with seizure freedom rates ranging from 63–80% [15–20]. However, there are numerous risks and variable long-term outcomes to hemispherotomy, the most likely being hemiplegia and homonymous hemianopia, contralateral loss of fine motor movement, and loss of language function with dominant hemisphere involvement [1]. Some patients may require additional surgeries or develop hydrocephalus as a complication. Dominant hemispheric surgery or surgery in older children with decreased neuroplasticity present special challenges with regards to weighing risks and benefits.

There are few articles in the literature exploring the impact of preoperative characteristics on long-term post-operative seizure outcomes [18, 21]. Recently, this issue was examined on a large scale via the Hemispherectomy Outcome Prediction Scale (HOPS) study using a combination of simple demographic, semiology, imaging, etiology, and prior surgical data [22]. An initial external validation trial of HOPS was unable to support its inceptive predictive value [23], and since all variables but the age at seizure onset in the scale are likely to remain constant among RE patients, it may not suffice for this population. Studies have analyzed trends of radiographic disease evolution, but biomarkers predictive of medical or surgical treatment success have not been identified [1, 7, 24]. Features, such as unihemispheric focal atrophy, peri-sylvian atrophy, and caudate involvement, are regularly seen in patients with RE, with later disease states typically demonstrating slowing of atrophic changes [1, 7]. Pathology, usually obtained during hemispherotomy, may also provide an estimate of disease severity post-surgery [25]. Based on our review of the literature, no other study has investigated preoperative imaging and post-operative pathology results in a combined way to help predict surgical outcomes. The goal of this study is to utilize our multicenter cohort to characterize if differences in pathology and/or imaging characterization of RE may provide a window into post-hemispherotomy seizure outcomes, which in turn could guide decision-making for parents and healthcare providers. 

## Methods

### Study design and data collection

This multi-institutional retrospective review of medical record, imaging, and pathology samples was approved by each individual institution’s review board. The institutions that participated in contributing data were Cincinnati Children’s Hospital, Seattle Children’s Hospital, and Washington University St. Louis Children’s Hospital. Data was collected from all known cases of peri-insular functional hemispherectomy for RE from the earliest available electronic medical records through January 2015. All patients were diagnosed with RE following the 2005 European consensus statement [14] as well as biopsy. A standardized database template was used to guide data collection from medical records. For Engel and ILAE classification status, the most recent available record describing the patient’s seizure burden was used.

All available historical cranial imaging for each patient was uploaded to a centralized PACS database and analyzed using a modified Bien staging system by a single neuroradiologist (JL) [26]. MRI analysis under the Bien staging system included assessment of unihemispheric cortical atrophy, gray, or white matter T2/FLAIR hyperintensities, and signal change or atrophy of the ipsilateral caudate head. Detailed commentary was also provided by retrospective review of all 3D imaging by the same neuroradiologist [7, 27]. Separate staging scores were administered for basal ganglia structures and cortical structures. Regional differences were noted in many cases such that a range of Bien scores could exist for each patient. Where preoperative positron emission tomography (PET) scans were available, these were also reviewed by the same radiologist and analyzed specifically for hypometabolism on a lobar basis including analysis of the cerebellar hemispheres.

Pathology slides and remaining block specimens were all sent for review by a single pathologist (CF) for staging using the system described by Robitaille and modified by Pardo et al. in 2004 [25, 28]. This staging system assesses histopathologic changes in a four-category progression that represent early, mid-, late-, and end-stage hemispheric destruction regionally [14, 25]. Pathology analysis was carried out separately in each available brain region from the provided specimens. Additionally, a single Robitaille/Pardo stage was assigned to each patient based on the predominate pathologic stage found within all available tissue for that patient.

### Statistical analysis

Relationships between categorical and continuous variables were analyzed with Microsoft Excel to determine Pearson correlation values and statistical significance was set at an alpha of < 0.05. *P* values were calculated with the data analysis regression tool in Excel.

## Results

### Demographics

Demographic data is highlighted in Table 1. A total of 27 patients were reviewed between the three hospitals. The participant population consisted of 12 male patients (44%) and 15 female patients. All patients had undergone hemispherotomy. Fourteen patients (52%) had surgery on the left hemisphere and 13 underwent surgery on the right hemisphere (48%). Twenty-six patients had follow-up data available for review. The mean follow-up time was 5 years (range: 1 month to 24 years). Preoperatively, ten patients (37%) had documented history of EPC. The mean age at surgery was 8.9 years (SD 4.0), and the average first seizure to surgery interval was 4.3 years (SD 3.9). Fourteen patients had reported post-operative seizure freedom (54%) after hemispherotomy at last follow-up.

**Table 1:** Demographics

Demographics	*N* (SD)
**Sex**	
Male	12
Female	15
Total	27
**Mean age seizure onset (yrs)**	4.7 (2.9)
**Mean age at surgery (yrs)**	8.9 (4.0)
**First ictus to surgery interval (yrs)**	4.3 (3.9)
**Site of surgery**	
Left	14
Right	13
History of EPC^*^	10
**Post op seizures**	
Yes	14
No	12
**Mean follow-up time (yrs)**	5 (5.3)

^*^EPC epilepsia partialis continua

### MRI and PET

The earliest available preoperative MRI datasets were used for analysis. Preoperative MRI imaging data were available in 19 patients (Table 2) looking at characteristics of both cortical and basal ganglia edema, signal changes, signal enhancement, diffusion restriction, and atrophy.

**Table 2:** Imaging findings

**MRI/PET findings**	***N***
**Cortical swelling**	12
Frontal	6
Parietal	2
Temporal	4
Insular	2
**Cortical signal**	14
Frontal	10
Parietal	3
Temporal	4
Insular	6
**Cortical atrophy**	7
Frontal	4
Insular	3
**Basal ganglia swelling**	3
**Basal ganglia signal**	6
Caudate	4
**Basal ganglia atrophy**	4
Caudate	4
**Diffusion restriction**	3
**PET scan**	
Normal	1
Hypometabolism	11
Left hemisphere	6
Right hemisphere	5

Twelve patients (68%) showed evidence cortical thickening/edema, most commonly in the frontal lobes (50%). The temporal lobe was the second most common location of cortical edema (33%). Basal ganglia (BG) edema was observed in three patients (16%).Increased cortical T2/FLAIR signal was a common finding in the sample population with 74% of patients showing some cortical signal change, occurring more frequently in the frontal lobes as well (71%). The insular (43%) region was also a common location of signal changes. BG signal changes were observed in six patients, 67% of which had caudate involvement. In contrasted MRI studies, there was no evidence of cortical enhancement. For diffusion analysis, three patients demonstrated restricted diffusion which was associated with various regions. Of the patients with observed diffusion restriction, all had evidence of cortical edema.

Seven patients with MRI data exhibited cortical atrophy (37%), comprising both the frontal (57%) and insular regions (43%). Four patients (21%) with MRI data showed signs of BG atrophy. Of the BG atrophic patients, all demonstrated preferential caudate involvement.

The highest recorded imaging staging for both the cortical and BG regions was recorded. Five percent of patients were cortical stage 1, 11% were stage 4, and 84% were grades 2–3. For BG staging, 37% of patients had no basal ganglia findings, 21% were stage 4, and 42% were stages 2–3. Furthermore, regression analysis was performed to determine if seizure duration (determined from first ictus to first recorded MRI) was associated with severity seen on MRI staging. There was a positive association between epilepsy duration and highest documented cortical severity (*r* = 0.46, *p* = 0.04).

Positive emission tomography (PET) data prior to surgical intervention was available for 12 patients (Table 2). One patient’s PET scan exhibited normal findings. Of the PET positive patients, all showed unihemispheric involvement concordant with the structural MRI. Despite diffuse hemispheric hypometabolism, two patients showed predominance within the frontal lobe (22%). Two patients (17%) demonstrated ipsilateral cerebellar hypometabolism and three (25%) had contralateral cerebellar hypometabolism Table 3.

**Table 3:** Pathology findings

Pathology findings	*N*
**Frontal**	
Stage 1	7
Stage 2	4
Stage 3	2
Stage 4	1
**Insular**	
Stage 1	5
Stage 2	0
Stage 3	1
**Temporal**	
Stage 1	10
Stage 2	3
Stage 3	4
**Hippocampal**	
Stage 1	4
Stage 2	2
Stage 3	0
**Hippocampal sclerosis**	6

Comparison where available for Bien imaging stages (initial diagnostic MRIs) and Robbitaille/Pardo pathology staging with last available ILAE class outcome score

### Pathology findings

Pathology slides were analyzed in 27 RE patients, all of which were obtained from peri-insular hemispherotomy. The pathology data is referenced in Table 4. Seventeen patients had positive temporal lobe findings (62.9%), ten of these patients were characterized as Robitaille/Pardo stage 1 (58.8%). Insular (22%) and hippocampal (22%) involvement was noted in six patients, with 83.3% and 66.6% favoring stage 1, respectively. Hippocampal sclerosis was seen in six patients (22.2%). Eight patients (29.6%) had only one pathology stage present through their available specimens. Ten patients (37%) demonstrated deviation of pathological severity greater than one stage across their available specimens.

**Table 4:** Participant imaging staging + pathology stages with associated ILAE seizure outcome

Participant	MRI—cortical stage	MRI—BG stage	Pathology stage	Pathology locations	Hippocampal sclerosis	ILAE class
CCHMC 1	3	0	1	Insula	Yes	1
CCHMC 2	2	0	2	Frontal, temporal, insula, hippo	No	5
CCHMC 3	2	0	1	Frontal, temporal	No	1
CCHMC 4	2	4	2	Frontal, temporal, insula	Yes	1
CCHMC 5	4	0	1	Frontal, temporal, insula	No	1
SCH 1	1	3	3	Frontal	No	1
SCH 2	2	2	1	Temporal	No	5
SCH 3	3	2	2	Temporal	No	1
SCH 4	3	3	2	Frontal	No	1
SCH 5	3	0	1	Insula	No	2
SCH 6	2	0	1	Insula	No	4
SLCH 1	4	4	1	Frontal, temporal	No	1
SLCH 2	3	3	1	Frontal, temporal, hippo	No	1
SLCH 3	3	3	1	Frontal, temporal	Yes	5
SLCH 4	2	0	1	Frontal, temporal, hippo	No	1
SLCH 5	3	3	2	Frontal, temporal, hippo	No	1
SLCH 6	3	3	1	Frontal, temporal	Yes	4
SLCH 7	3	4	1	Temporal	No	1

*BG* basal ganglia, *hippo* hippocampus

### Imaging and pathology correlation studies

Imaging staging from the cortex and BG were compared with pathology stages and post-operative seizure outcome data, which was organized per individual participant (Table 4). Correlation analysis of the pathology and imaging data can be seen in Table 5. It was hypothesized that pathology Pardo staging data might correlate with Bien imaging staging. This analysis determined there was no statistically significant connection between general pathology staging to either highest or lowest cortical or basal ganglia imaging staging between patients. Of note, the strongest correlation showed a positive association between highest pathological staging to highest MRI staging (Pearson *r* = 0.43). However, this association was still not statistically significant (*p* = 0.07).

**Table 5:** Imaging vs. pathology correlations

Correlation	*N*	Pearson *r*	*p*-value
Pathology vs. MRI stage (cortical/low)	19	−0.28	0.25
Pathology vs. MRI stage (cortical/high)	19	−0.26	0.28
Pathology vs. MRI stage (BG/low)	18	0.25	0.33
Pathology vs. MRI stage (BG/high)	19	0.39	0.10
Highest pathology vs. highest imaging	19	0.43	0.07
Pathology vs. highest imaging	19	0.14	0.57

*BG* basal ganglia

### Seizure outcomes

Seizure outcomes were compared to multiple preoperative and intra-operative measures. A comprehensive list can be viewed in Table 6. Comparing the time interval of first ictus to surgery did not predict seizure outcomes (*p* = 0.42 ILAE). Similarly, the patient’s age at time of surgery had no correlation to seizure outcomes (ILAE *p* = 0.87). General pathological stage also failed to predict seizure outcomes (ILAE *p* = 0.55). Comparing seizure outcomes to MRI staging (cortical or BG) data yielded no significant predictive power.

**Table 6:** Seizure freedom correlations

Correlation	*N*	Pearson *r* (Engel)	*p*-value (Engel)	Pearson *r* (ILAE)	*p*-value (ILAE)
Ictus to surgery interval vs. seizure outcome	24	0.23	0.27	0.17	0.42
Age at surgery vs. seizure outcome	24	0.06	0.77	0.04	0.87
Age at first ictus vs. seizure outcome	24	0.35	0.09	0.30	0.15
Seizure outcome by MRI stage (cortical/high)	20	− 0.24	0.30	− 0.26	0.26
Seizure outcome by MRI stage (BG/high)	20	0.08	0.74	0.02	0.94
Seizure outcome by path stage (overall)	23	− 0.14	0.52	− 0.13	0.55
Seizure outcome by path stage (high)	22	− 0.20	0.37	− 0.19	0.34
Seizure outcome by path stage (low)	23	− 0.21	0.33	− 0.19	0.38
Seizure outcome by highest path + highest MRI (BG + cortical)*	19	− 0.30	0.21	− 0.32	0.19
Seizure outcome by lowest path + lowest MRI (BG + cortical)*	18	− 0.38	0.13	− 0.37	0.13

^*^Summated pathology, basal ganglia (BG), and cortical imaging staging

HOPS scores were obtainable in 25 patients. Fourteen patients demonstrated a HOPS score of 1 (> 3.5 years old at seizure onset). No patients met the criteria for a HOPS score of 3 (< 3 months at seizure onset). All other patients were given a score of 2 (*n* = 11). Of the 14 participants with documented seizure freedom, ten exhibited a HOPS score of 1 (71%), the remaining demonstrated a HOPS score of 2 (29%).

Additive analysis combining both pathology and MRI imaging staging compared to seizure outcomes was performed as well. Our sample size precluded us from performing a multivariate analysis. Instead, pathologic staging, cortical imaging, and BG imaging staging were analyzed through a combined score analysis. Both the summated highest and lowest values of these categories were considered. A combination score was created by adding either the highest or lowest MRI scores from both basal ganglia and cortex in addition to highest or lowest regional pathology score for each patient (Table 5). Both the lowest and highest pathology plus MRI combined scores suggest that, as with pathology scores alone, more severe total scores trend toward better outcomes (*r* =  − 0.30 to − 0.38). These combined scores trended toward slightly stronger correlations and lower *p* values than independent comparisons of pathology or imaging to outcomes. However, these findings were also not statistically significant.

## Discussion

RE remains an incompletely understood and devastating neurologic disorder demanding the most aggressive of neurosurgical techniques to treat. Fortunately, many patients can achieve a state of prolonged or permanent seizure freedom while maintaining good functional neurologic performance with functional hemispherotomy. The seizure freedom data from this study was approximately 54%. A previous meta-analysis has reported seizure outcomes for Rasmussen’s treated with hemispherotomy ranging from 17 to 100% with the 5-year mean at 65% [29]. Given the morbidity of the hemispherotomy procedure, it would be very helpful to determine which RE patients are more likely to have robust seizure freedom outcomes for surgical selection and prognostication. Currently, clinical and radiographic features have not been particularly useful tools for predicting seizure freedom rates on an individual patient basis. A recent effort to address this issue has been made for all hemispherectomy patients in the form of the HOPS scale, but this currently lacks external validation and largely boils down to the single variable of age at seizure onset for RE patients. Applying the HOPS to our multicenter RE patient cohort, we noted lower seizure freedom percentages than study reference rates (71% vs. 96% for a score of 1, 45% vs. 92% for a score of 2) [22]. The trend in outcome prediction correlates, but the tool does not appear calibrated to make accurate predictions in the RE subset of hemispherotomy patients. We sought to combine retrospective data with a focus on radiographic and pathologic features to determine if criteria from these data could assist with predicting seizure outcomes. Specifically, we investigated if there might be predictive value in combining both radiographic findings and pathology findings. If a useful biomarker was found, this could potentially justify pre-hemispherotomy biopsies for prognostic purposes. Ultimately, our analysis did not find evidence for combined analysis of imaging and pathology staging being predictive for seizure outcomes. However, there were independent trends in both imaging and pathology findings that are noteworthy.

### Radiographic findings

With a focus on examining the earliest available MRI scans by a single neuroradiologist to detect potential early prognostic features, there was abundant variability in the scans. Nonetheless, some descriptive themes arose from this analysis. The images showed a predilection for abnormalities in the superior frontal or medial frontal gyri. The imaging at the time of presentation typically showed the most acute radiographic appearance consistent with early immune-mediated inflammatory response [30, 31]. The hippocampus on the affected side was typically abnormal in appearance, and PET imaging was abnormal on the affected hemisphere. Caudate signal change or caudate atrophy was not always present in this pediatric dataset (42%) but was on par with other studies (38–89%) [7, 9, 31, 32]. The putamen was about half as likely to show atrophy compared to the caudate and much less likely to signal changes. The frequency of putaminal signal changes exhibited in this cohort are consistent with prior studies, but putaminal atrophy findings (11%) were less common than previously described (25–79%) [7, 32]. No cases showed enhancement and diffusion restriction was rare. Diffusion restriction is not a typical feature of RE [7, 33, 34] but has been reported with early-stage edema [35]. The three cases observed with diffusion restriction all demonstrated cortical edema as well.

### Pathology findings

The widespread variability of pathologic changes within individual patients makes single point biopsy potentially misleading with regard to the overall severity of the cellular impact of the RE disease process. Our review of multi-institutional pathological specimens by a single neuropathologist confirmed widespread variability of Robitaille/Pardo staging within and across patients [25]. While pathology has been shown to provide clues to the duration or progression of illness based on stage and location, it does not appear to hold significant predictive power with regards to the long-term success of disconnective surgical intervention [25]. A prior study has shown that younger age and shorter time between diagnosis and surgical treatment are linked to greater durations of seizure freedom [29]. These are likely proxies of decreased pathological burden. With a mean post-operative follow-up assessment of 5 years, we did not see a correlation between either age at surgery or pathological burden and seizure freedom rate.

Limitations of this study include the retrospective nature of data collection, variability in available follow-up data, unavailability of pathology and/or imaging data for all patients, inconsistent pathology sampling at the time of surgeries, and a prolonged period of data collection.

For many patients with Rasmussen’s disease, the only option for seizure control is hemispheric disconnection surgery and many pediatric patients achieve complete seizure freedom with good functional outcomes. However, long-term surgical seizure outcomes have only modestly improved in recent decades and improvements in patient selection for these surgeries could aid surgical decision-making. While combining currently available MRI imaging findings and tissue pathology data does not appear to help with surgical outcome prognostication, other biomarkers should still be explored. RNA expression levels of interferon genes [36] or CSF protein markers [37] might be better variables to combine with imaging findings for multivariate prediction of hemispherotomy seizure outcomes. Since the underlying etiology of RE is incompletely understood, it is possible that the observed phenotype may have different variants in mechanisms that drive the hemispheric encephalitis. It is also possible that the disease process lies on a spectrum. Here, we have described some of the more commonly observed radiographic and pathologic findings in our multi-institutional cohort. We did observe a trend toward the cases that were at the more severe end of the radiographic and pathologic spectrum having the best response to hemispherotomy. Neither the patient age at surgery nor the duration between seizure onset and surgical intervention affected the seizure outcomes. Given the current lack of reliable indicators for long-term outcomes, we currently advocate for disconnective surgery at an early age such that additional treatment routes are not delayed if seizures are refractory and the greatest opportunity for functional plasticity is present.

## Conclusion

Existing techniques for preoperative predictions of seizure freedom for hemispherotomy have not been shown to be effective in the setting of RE. Our analysis did not find evidence for combined analysis of imaging and pathology staging being predictive for seizure outcomes, prompting the need for other biomarkers to be explored.

## Data Availability

Data is available upon request by contacting the corresponding author.

## Declarations

### Statement of exclusivity

This manuscript is a unique submission and is not being considered for publication, in part or in full, with any other source in any medium. 
